# Association of the use of psychotropic drugs with hospitalization, cardiovascular events, and mortality in patients with type 2 diabetes: a propensity score-matched cohort study

**DOI:** 10.3389/fcdhc.2023.1181998

**Published:** 2023-07-05

**Authors:** Hidetaka Hamasaki, Hidekatsu Yanai

**Affiliations:** ^1^ Hamasaki Clinic, Kagoshima, Japan; ^2^ Department of Diabetes and Endocrinology, National Center for Global Health and Medicine, Kohnodai Hospital, Ichikawa, Japan

**Keywords:** psychotropic drugs, type 2 diabetes, hospitalization, handgrip strength, skeletal muscle, psychiatric comorbidity

## Abstract

**Background:**

Use of psychotropic drugs (PD) may be associated with impairment of physical function. However, few studies have assessed the impact of PD on health outcomes in patients with type 2 diabetes. This study aimed to examine the associations between psychotropic drug use and handgrip strength (HGS) and between the use of PD and hospitalization in patients with type 2 diabetes.

**Methods:**

From April 2013 to December 2015, we conducted a retrospective cohort study in patients with type 2 diabetes at the National Center for Global Health and Medicine Kohnodai Hospital. Patients aged 20 years and over who can measure HGS were included. All participants received nutritional guidance regarding diet therapy for type 2 diabetes at baseline. Nonpsychotropic drug users were matched one-to-one with the PD users using propensity score matching method with respect to their baseline covariates. The differences in HGS and the number of patients who had hospitalizations during the study period were examined. By Cox proportional hazard regression analysis, the association between the use of PD and repeated hospitalizations was estimated.

**Results:**

A total of 1,282 patients were enrolled and followed up for 2.36 ± 0.73 years. In the propensity score matching cohort, HGS was significantly lower (p = 0.006) in PD users than non-PD users. PD users had more hospitalizations than non-PD users. Cox proportional hazard regression analysis confirmed the association of repeated hospitalizations with the use of PD (hazard ratio = 2.138; 95% confidence interval, 1.144–3.995, p = 0.017)). In addition, HGS was significantly and inversely correlated with the number of hospitalizations (r = −0.143, p = 0.013).

**Conclusions:**

The use of PD could increase the risk of repeated hospitalizations. Skeletal muscle may play a role in reducing the risk of hospitalization in patients who are treated with PD.

## 1 Introduction

Patients with diabetes mellitus may suffer from psychological disorders, such as depression, anxiety, eating disorders, and schizophrenia (1). Although the definition of psychological disorders/syndromes in patients with diabetes varies across clinical studies, ranging from self-reported symptoms to using formal diagnostic criteria such as Diagnostic and Statistical Manual of Mental Disorders, interventions for psychological problems in patients with diabetes result in an improvement in the management of diabetes (1). Chronic insomnia with a sleep duration ≤ 5 h has been associated with an increased risk of diabetes (2). A meta-analysis showed that depression was associated with a 60% increase in the risk of development of type 2 diabetes (3). The prevalence of type 2 diabetes is 10% in patients with schizophrenia, and the relative risk of developing diabetes is 2.5 times higher in patients with schizophrenia than that in the general population (4). Therefore, clinicians should pay attention to such psychiatric comorbidities in patients with diabetes.

Antipsychotic drugs probably increase the risk of diabetes (5, 6) through causing obesity (7) and a reduced insulin sensitivity (8). Furthermore, although psychotropic drugs (PD) are widely used even in the absence of a confirmed diagnosis of psychiatric disorders, epidemiological studies have shown that antipsychotic drug use is associated with an increased mortality risk in patients with Parkinson’s disease (9) and Alzheimer’s (10). In addition, high-dose benzodiazepines use has a dose–response relationship with mortality in patients with schizophrenia (11); similarly, antipsychotic drug use in combination with benzodiazepines is associated with an increased risk of mortality in patients with dementia (12). Furthermore, a systematic review and meta-analysis has shown that antipsychotics, antidepressants, and benzodiazepines were consistently associated with a higher risk of falls (13). On the other hand, the relationship between the use of benzodiazepines, antidepressants, and antipsychotics and serious diseases, such as pneumonia, cancer and cardiovascular (CV) disease is controversial (14–18). In the literature, few studies have assessed the impact of PD on such health outcomes in patients with type 2 diabetes.

Generally, patients with type 2 diabetes have a lower energy expenditure, physical activity duration (19), and muscle strength (20, 21) than healthy individuals. Recently, we showed that handgrip strength (HGS), which is a simple and cost-effective method for evaluating muscle strength, predicts hospitalization, occurrence of CV events, and death among Japanese patients with type 2 diabetes (22). van Milligen et al. reported that women with depression and anxiety disorder had lower HGS compared with healthy controls (23). Antipsychotic drugs also decrease physical activity, whole body balance, and cardiorespiratory endurance (24). We hypothesize that PD, including benzodiazepine, antidepressant, and antipsychotic use, is associated with impairment of physical function such as HGS and subsequently leads to an increased risk of hospitalization. Thus, in this study, we examined the association of PD use with HGS and deaths, CV events, and hospitalization in patients with type 2 diabetes.

## 2 Materials and methods

### 2.1 Study design and subjects

**Figure 1:**
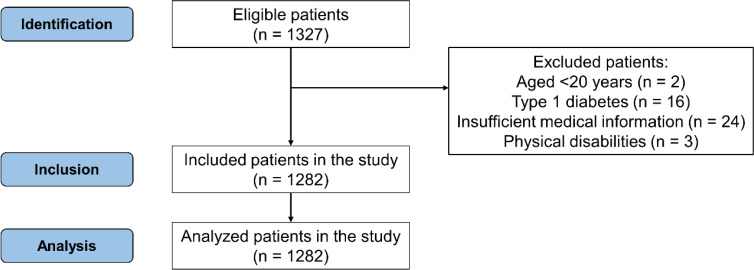
STROBE flow chart.

We conducted a retrospective cohort study in patients with type 2 diabetes who were treated at the National Center for Global Health and Medicine Kohnodai Hospital between April 2013 and December 2015. Baseline is the date on which each patient’s data, such as medical history, anthropometric, physiological, and biochemical data, were collected. A total of 1,327 individuals aged >20 years with type 2 diabetes whose medical history, i.e., regular treatment with benzodiazepines, antidepressants, and antipsychotics, was collected at first examination were eligible for inclusion in our analyses. Patients aged <20 years (n = 2) with type 1 diabetes (n = 16) and without medication information (n = 24) were ineligible for inclusion. Additionally, patients whose HGS could not be measured because of disabilities, such as cerebral infarction sequelae (n = 3), were also excluded (**Figure 1**). The PD patient group was defined as follows (1): Patients who were prescribed at least one PD (benzodiazepines, antidepressants, and antipsychotics) at the first examination during the study period; (2) Patients who continuously received PD and confirmed medication adherence during the study period. Patients who were not prescribed PD at the start of follow-up did not receive any new PD during the course of the study.

The study protocol was approved by the Medical Ethics Committee of the National Center for Global Health and Medicine (Reference No. NCGM-G-002052), and the study was performed in accordance with the Declaration of Helsinki.

### 2.2 Study procedures

At the first clinical visit, patients were instructed to consume a calorie-restricted diet of 25–30 kcal/kg (ideal body weight) each day by certified nutritional educators as diet therapy for diabetes and to continue the diet during the study period. The dietary adherence of patients was confirmed on every consultation day on a monthly basis. All patients were evaluated and followed up until death or at the end of follow-up in May 2016. At the end of the follow-up, the information on hospitalization was collected from medical record review. Next, the number of hospitalizations was calculated in all subjects, and hospitalization for two or more times was defined as repeated hospitalization. In other words, repeated hospitalization refers to any hospitalization that occurred after the initial hospitalization during the study period.

### 2.3 Anthropometric and physiological measurements

Participants’ height was measured using a rigid stadiometer (TTM stadiometer; Tsutsumi Co., Ltd., Tokyo, Japan). Their weights were measured using calibrated scales (AD-6107NW; A&D Medical Co., Ltd., Tokyo, Japan). Body mass index (BMI) was calculated as body weight in kilograms divided by the square of the body height in meters. Waist circumference was measured with the participant in a standing position at the level of the umbilicus at the end of exhalation. HGS was measured twice using a Smedley analog hand dynamometer (No. 04125; MIS, Tokyo, Japan) using both hands in a standing position. We used the average HGS in kilograms in our final analyses. Blood pressure was measured with the participant in a seated position using an automatic sphygmomanometer (HBP-9020; Omron Co., Ltd, Tokyo, Japan).

### 2.4 History taking and physical activity assessment

To collect participants’ baseline characteristics, trained technicians at the Clinical Research Center of the National Center for Global Health and Medicine at Kohnodai Hospital asked participants at the outpatient clinic about their physical activity levels, smoking and drinking habits, sleep duration, and medication use. The Brinkman index (number of cigarettes per day multiplied by the number of years) was calculated to quantify patients’ smoking habits (25). Using patients’ regular exercise habits, we calculated the exercise time per day based on exercise sessions per day × exercise duration per session.

### 2.5 Blood assessments

Blood samples were taken from the antecubital vein at the enrolment when HGS was measured. We measured plasma hemoglobin A1c (HbA1c) by high-performance liquid chromatography (HA-8180; Arkray, Tokyo, Japan). We calculated estimated glomerular filtration rate (eGFR) using the revised equation adjusted for the Japanese population (26).

### 2.6 Sample size

Sample size calculation was performed using G*Power (https://www.psychologie.hhu.de/arbeitsgruppen/allgemeine-psychologie-und-arbeitspsychologie/gpower). Our sample size had sufficient power to detect statistical significance (**Supplementary File**).

### 2.7 Statistical analysis

Continuous variables were expressed as the mean ± standard deviation (SD). Categorical variables were expressed as numbers and patient groups compared using χ2 test. Student’s t test or the Mann–Whitney test, depending on whether the variables followed normal or nonnormal distribution, was performed to detect significant differences between patients treated with PD and those treated without PD, as appropriate. Additionally, the relationship between the number of hospitalizations and HGS (in kilograms) was assessed using Spearman’s rank correlation coefficient. We also compared patient outcomes i.e., hospitalizations, CV events, and deaths for patients treated with and without PD before and after propensity score matching using χ^2^ tests.

A propensity score-matched analysis was performed to balance the characteristics of subjects at baseline between groups. Covariates were age, gender, BMI, alcohol consumption, exercise time, sleep duration, systolic blood pressure, and HbA1c levels. Based on the propensity score, PD users were matched to non-PD users by the nearest neighbor matching that is based on the greedy matching algorithm at a 1:1 ratio to create a propensity score-matched cohort (27). Subjects were matched based on the logit of the propensity score using a caliper width of 0.2 of SD. Standardized differences in each variable were calculated to confirm the balance between groups. In addition, the c-statistic for evaluating the goodness of fit was calculated. Subjects were then compared based on number of hospitalizations, CV events, and deaths.

Subsequently, Cox proportional hazard regression analysis was performed to assess the independent associations of mortality, CV events, and hospitalization with the use of PD in propensity score matched cohorts. Furthermore, multiple regression analysis was performed to assess relationship between hospitalization and HGS and use of PD.

P values of <0.05 determined by performing a two-sided test were considered statistically significance. Statistical analyses were performed using SPSS version 25 (IBM Co., Ltd., Chicago, IL).

## 3 Results

### 3.1 Participant characteristics prior to propensity score matching

This study enrolled 1,282 patients (709 men and 573 women) with type 2 diabetes. Of these, 379 (29.6%) patients were treated with PD, whereas 903 (70.4%) patients did not receive PD. A total of 314 patients (24.5%) received benzodiazepines, antidepressants, and/or antipsychotics; 175 (13.7%) received benzodiazepines; 93 (7.3%) received antidepressants; and 168 received antipsychotics (13.1%). The mean age and BMI of patients treated with PD were 60.1 ± 14.4) years and 26.9 ± 6 kg/m2, respectively. Patients with PD consume more alcohol (14.5 ± 26.7 g/day), engage in less exercise (9.5 ± 33.1 min/day), and sleep longer (7.8 ± 2.1 hours) compared to patients without PD. Additionally, patients with PD had lower systolic blood pressure (129.2 ± 18.5 mmHg), HbA1c levels (7.1 ± 1.4%), and HGS (22.3 ± 9.7 kg) compared to those without PD. Patients’ characteristics are listed in **Supplementary Table 1**.

Age, duration since diagnosis of diabetes, alcohol consumption, exercise time, systolic blood pressure, HbA1c, and HGS were lower in patients treated with PD than in those treated without PD. In contrast, BMI, sleep duration, and eGFR were higher in patients treated with PD. Patient groups did not differ by smoking status and diastolic blood pressure.

### 3.2 Participant characteristics after propensity score matching

After a propensity score-matched analysis, two groups of 254 well-matched patients were generated. The c-statistic was 0.717 (95% confidence interval [CI], 0.683–0.750), suggesting that the performance of the propensity score-matched model was acceptable. Of these, 76 patients (29.9%) received benzodiazepines only; 27 patients (10.6%) received antidepressants only; and 44 patients received antipsychotics (17.3%) only. Patient’s characteristics at baseline were balanced (**Table 1**); however, HGS was still lower in patients treated with PD that in those treated without PD.

**Table 1:** Characteristics of patients with or without psychotropic drugs in the matched cohort.

Characteristics	With psychotropic drugs	Without psychotropic drugs	p	Standardized difference
N	254	254	–	–
Age (years)	60.1 (14.1)	59.7 (14.9)	0.79	0.024
Gender (male/female)	129/125	129/125	1	<0.001
BMI (kg/m^2^)	26.3 (5.9)	26.4 (6.2)	0.84	0.018
Duration of diabetes (years)	10.5 (10.5)	9.3 (10.3)	0.21	0.112
Smoking habits (Brinkman index)	298.5 (441.1)	287.1 (557.1)	0.8	0.023
Drinking habits (g/day in ethanol consumption)	17.6 (30.2)	19.2 (31.2)	0.54	0.055
Exercise time (min/day)	9.5 (36.2)	8.6 (26.1)	0.75	0.028
Sleep duration (h)	7.5 (2.1)	7.5 (1.7)	0.98	0.003
Systolic blood pressure (mmHg)	130.4 (17.7)	131.1 (21.2)	0.68	0.036
Diastolic blood pressure (mmHg)	74 (13.6)	74.8 (14.4)	0.53	0.056
HbA1c (%)	7.4 (1.6)	7.4 (1.6)	0.96	0.005
eGFR (mL/min/1.73m^2^)	76.1 (22.3)	76 (23.9)	0.98	0.003
Handgrip strength (kg)	22.7 (9.3)	25.1 (10.2)	0.006	0.243

Data are represented as the mean value (SD) except for the number of subjects and sex. BMI, body mass index; HbA1c, hemoglobin A1c; eGFR, estimated glomerular filtration rate.

### 3.3 Patient outcomes

During a mean follow-up of 861 ± 265 days, 9 patients (1.8%) died, 4 (0.8%) experienced CV events, and 336 (66.1%) were admitted to our hospital in the matched cohort. All deceased patients had one or more hospitalizations during the study period. Among the PD group, 6 patients died, one experienced CV events and 119 were admitted. The total number of hospitalizations was 482. Of these, 185 (38.4%) were in the Diabetes and Endocrinology ward, 81 (16.8%) were in the Surgery ward, 55 (11.4%) were in the Internal Medicine ward, 34 (7.1%) were in the Hepatology ward, 27 (5.6%) were in the Gastroenterology ward, 24 (5.0%) were in the Ophthalmology ward, and 40 (8.3%) were in the Psychiatry ward. No significant difference in hospitalization was observed between groups; however, the number of patients treated with PD who were admitted to our hospital more than once was significantly higher than those treated without PD (**Table 2**).

**Table 2:** Comparison of health outcomes between patients with or without psychotropic drugs in the full and matched cohorts.

	Full cohort			Propensity score matched cohort		
Outcomes	With psychotropic drugs	Without psychotropic drugs	p	With psychotropic drugs	Without psychotropic drugs	p
Hospitalizations	168	389	0.71	119	117	0.93
Repeated hospitalizations	75	149	0.17	54	36	0.048
Cardiovascular events	2	12	0.25	1	3	0.62
Deaths	8	12	0.33	6	3	0.50

Moreover, Cox proportional hazard regression analysis confirmed the association of repeated hospitalizations (three or more times) with the use of PD (hazard ratio [HR] = 2.138; 95% CI, 1.144–3.995, p = 0.017), while there are no significant associations between the use of PD and all-cause mortality and CV events (**Table 3**).

**Table 3:** Cox proportional hazard regression analysis for evaluating the associations of the use of psychotropic drugs with all-cause mortality, cardiovascular events, hospitalization, and repeated hospitalizations in patients with type 2 diabetes.

	All-cause mortality			CV events			Hospitalization (once)			Repeated hospitalization (two or more times)			Repeated hospitalization (three or more times)		
	HR	95% CI	p	HR	95% CI	p	HR	95% CI	p	HR	95% CI	p	HR	95% CI	p
Use of PD	1.814	0.452–7.277	0.4	0.93	0.72–1.2	0.58	0.31	0.032–3.005	0.31	1.366	0.896–2.083	0.15	2.138	1.144–3.995	0.017

CV, cardiovascular disease; HR, hazard ratio; CI, confidence interval; PD, psychotropic drugs.

**Figure 2:**
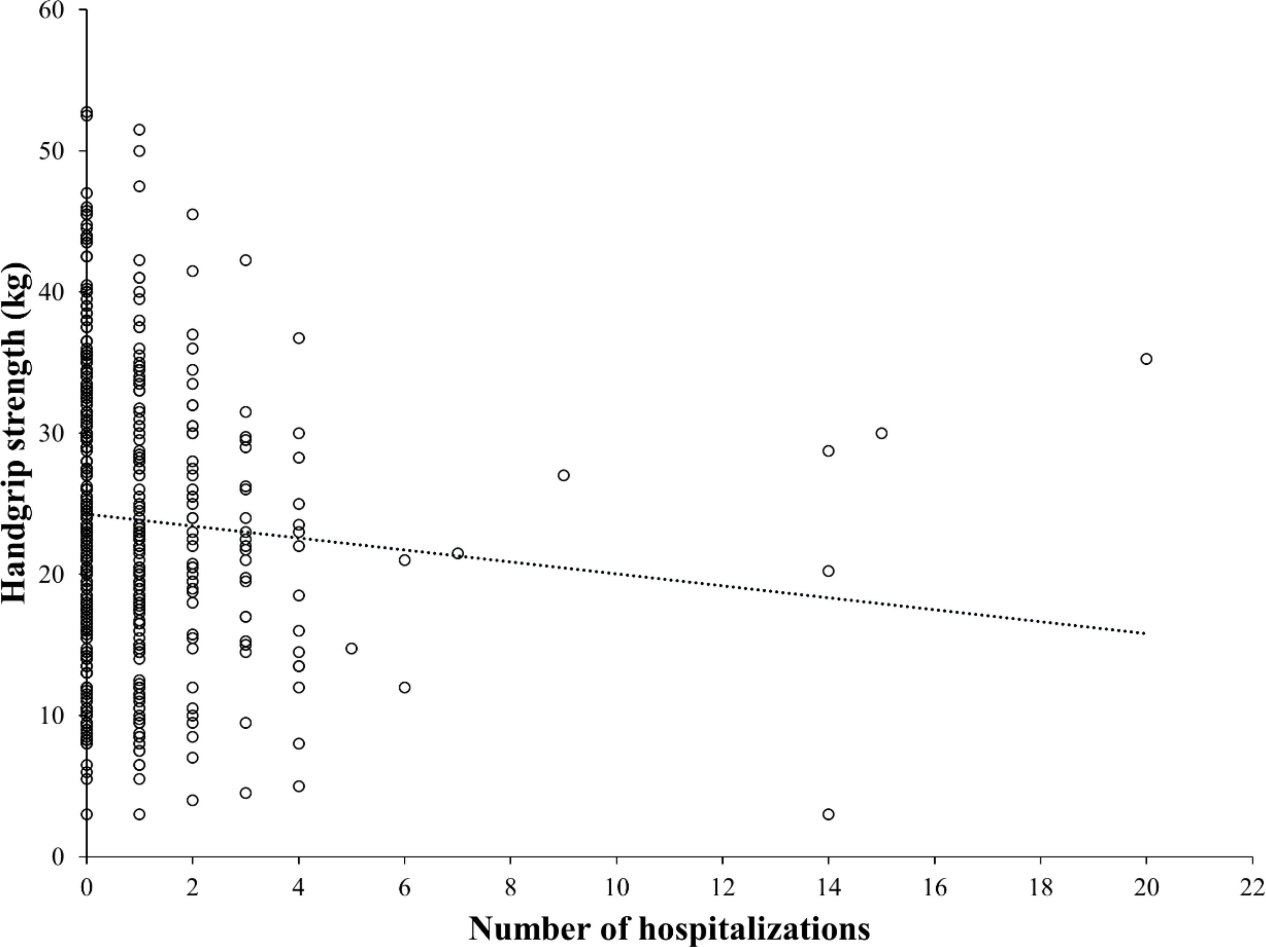
Correlation coefficient between handgrip strength and number of hospitalizations.

There is a negative correlation between HGS and the number of hospitalizations (r = −0.143, p = 0.013) (**Figure 2**). Furthermore, multiple regression analysis identified a positive association between repeated hospitalizations (three or more times) and the use of PD (β = 0.119, p = 0.007) (**Table 4**).

**Table 4:** Multiple regression analysis for evaluating the associations of the use of psychotropic drugs with hospitalization and repeated hospitalizations in patients with type 2 diabetes.

	Hospitalization (once)		Repeated hospitalizations (two or more times)		Repeated hospitalizations (three or more times)	
	β	p	β	p	β	p
Psychotropic drugs	–0.01	0.82	0.083	0.062	0.119	0.007
Handgrip strength	–0.15	0.001	–0.079	0.078	–0.082	0.065

## 4 Discussion

The main aim of this study was to examine the association of PD use with HGS and hospitalization in patients with type 2 diabetes. We demonstrated that PD users had lower HGS and more hospitalizations in a type 2 diabetes population than non-PD users. To the best of our knowledge, this study is the first to demonstrate that patients with type 2 diabetes receiving PD have decreased HGS, and such medication is associated with repeated hospitalizations in patients with type 2 diabetes.

No significant association between the use of PD and all-cause mortality was observed in this study; however, hospitalization and death share a commonality in indicating a decline in physical function. The average number of hospitalizations was higher in deceased patients than in surviving patients (3.44 ± 4.1 times vs. 0.88 ± 1.78 times, p <0.001 by Mann-Whitney U test). The number of repeated hospitalizations was also higher in deceased patients than in surviving patients (two or more times; p = 0.001; three or more times; p = 0.006 by χ^2^ test). In this study, six out of nine deceased patients were treated with PD, suggesting that the inappropriate use of PD could cause serious harm to physical health.

### 4.1 Psychotropic drugs and skeletal muscle

Cross-sectional studies have shown that female patients with depressive or anxiety disorders had lower HGS (23) and patients with schizophrenia have more impairments in muscular strength, endurance, and flexibility compared with healthy controls (28). In addition, higher antipsychotic dosages were associated with poor physical function (28), and benzodiazepines were found to increase the risk of fall by muscle relaxant effect in older adults (29). The findings of previous studies suggest that psychotropic medication decreases muscle strength and physical fitness.

Although the causal relationship between physical function and psychotropic medication is unknown, benzodiazepines, antidepressants, and antipsychotics may induce muscle weakness and increase the risk of hospitalization. Recently, Sandvik et al. (30) reported that the use of PD was significantly associated with reduced handgrip strength in older hospitalized patients.

We cannot reveal the mechanism underlying the unfavorable impact of PD on muscle strength and physical function based on the findings of this study alone; however, PDs have the possibility of damaging skeletal muscle. PDs have a variety of adverse health effects, such as dizziness, drowsiness, unconsciousness, fatigue, and sleep disturbances. In addition, benzodiazepines are well known as having skeletal muscle relaxant effects (31). Although the underlying mechanism is unclear, use of antipsychotics is associated with the elevation of creatinine kinase and rhabdomyolysis (32). Furthermore, a benzodiazepine, namely, diazepam that enhances the activity of the GABA_A_ receptors increases muscle sympathetic nerve activity and blood pressure during handgrip exercise in humans (33). Sympathetic nerve and arterial blood pressure responses to exercise is exaggerated in type 2 diabetes (34); thus, patients with type 2 diabetes might be prone to adverse effects of benzodiazepines. Such sympathetic nervous dysfunction decreases skeletal muscle blood flow, which might cause muscle weakness (35).

### 4.2 Hospitalization due to the use of psychotropic drugs

To emphasize, few studies have examined the association between PD and hospitalization in patients with diabetes. An observational cohort study in a nursing facility reported that psychotropic and psychoactive drugs were associated with an increase in the rate of hospitalization; however, the reasons for hospitalization were various, and how such drugs affect the risk of hospitalization was not clarified (36). However, hypoglycemia may also be associated with reduced physical fitness, which results in the increased risk of hospitalization in patients with PD. Indeed, the use of antipsychotics is significantly associated with an increased risk of hypoglycemia in older adults (37). Severe hypoglycemia is strongly associated with all-cause mortality (HR = 2.69; 95% CI, 1.97 to 3.67), cardiovascular mortality (HR = 2.68; 95% CI, 1.72 to 4.19), and other health outcomes, including cancer and respiratory and digestive diseases (38). Ogama et al. (39) reported that glucose fluctuations were independently and significantly associated with low HGS and muscle mass after adjusting for HbA1c levels. Ørngreen et al. (40) showed that decreased muscle mass could increase the risk of hypoglycemia in patients with neuromuscular disease. Skeletal muscle is an important source of gluconeogenesis in the fasting state and plays a crucial role in the regulation of glucose homeostasis (41). In this study, patients with PD might have experienced hypoglycemia due to decreased muscle fitness, resulting in some impairment of physical function and hospitalizations. However, we did not investigate whether study participants experienced hypoglycemia during the study period. Therefore, further investigations are warranted.

### 4.3 Limitations

Some limitations need to be addressed in the present study. First, we enrolled subjects who regularly received psychotropic medications; however, we did not investigate whether they were diagnosed with mental disorders, such as schizophrenia, depression, bipolar, and anxiety disorder. Thus, our findings cannot refer to the association of mental disorders with hospitalization in patients with type 2 diabetes. However, the number of patients admitted to the psychiatric ward was small (n = 16) during the 3-year follow-up, suggesting that the number of patients with severe mental disorders was relatively few in this study cohort. However, this issue is the most critical when assessing the effect of PD other than the mental illness itself. Further studies which examine the relationship of health outcomes with the use of PD and the existence of mental disorders separately are required. Second, the matched cohort is limited by small sample size and relatively short follow-up period to identify the significant difference in CV events and deaths between groups and limited generalizability. Considering that psychiatric disorders are common in patients with diabetes (42), multi-institutional or population-based studies may be required to obtain a larger cohort. Third, we did not investigate detailed causes of hospitalizations (e.g., name of disease, severity of disease); therefore, how the use of PD was associated with hospitalization is unknown. Finally, we grouped benzodiazepines, antidepressants, and antipsychotics together as PD in this study; however, each drug class should be investigated separately in future studies. Each drug has different physical and mental effects depending on the type of drug. Not all PDs, but second-generation antipsychotics, cause weight gain and reduce insulin sensitivity and glucose tolerance, which may lead to the development of type 2 diabetes (43). Antidepressants may exert a cardioprotective effect and reduce the risk of CV events (18). In this study, 27 patients took only antidepressants, which could have possibly affected the study results. Despite these limitations, our findings suggest that the use of PD leads to unfavorable health outcomes in patients with type 2 diabetes.

### 4.4 Suggestions for future studies

Randomized controlled trials (RCTs) are considered the gold standard in evidence-based medicine research. However, there are situations where conducting such trials may not be feasible or ethical, necessitating the reliance on observational studies. It is unethical to investigate the effects of PD on hard endpoints, such as death or CV events, in patients who require medication through RCTs. In this context, propensity score matching, as used in this study, is a practical method for estimating causal effects in observational studies (44). Nevertheless, there may still be unknown and unmeasured confounding factors, despite the use of appropriate methods. Therefore, future studies should incorporate additional information about study participants, including the presence or absence of mental disorders, detailed disease conditions, educational level, socioeconomic status, and genetic information. For instance, pharmacogenetic variants have a significant impact on the metabolism of PD, and genetic testing is considered crucial in determining whether the use of PD is toxic or therapeutic for patients (45). Furthermore, the interactions between PDs should also be taken into account, as a high number of PD interactions can result in severe health issues in clinical practice (46). Ideally, researchers should examine both the individual and interaction effects of PD on health outcomes. Well-designed future studies of this nature are warranted.

In conclusion, the use of PD could increase the risk of repeated hospitalizations in patients with type 2 diabetes. An increase skeletal muscle strength may reduce the risk of hospitalization in patients treated with PD. Our findings suggest that clinicians should judiciously prescribe PD to patients with type 2 diabetes.

## Data availability statement

The raw data supporting the conclusions of this article will be made available by the authors, without undue reservation.

## Ethics statement

The studies involving human participants were reviewed and approved by The Medical Ethics Committee of the National Center for Global Health and Medicine. Written informed consent for participation was not required for this study in accordance with the national legislation and the institutional requirements.

## Author contributions

HH conducted the study, performed data analyses, drafted and revised the manuscript. HY critically reviewed the manuscript and the scientific interpretations of study results. All authors read and approved the final manuscript.
