# Supplementary material for: Association of the use of psychotropic drugs with hospitalization, cardiovascular events, and mortality in patients with type 2 diabetes: a propensity score-matched cohort study
Source: Front Clin Diabetes Healthc. 2023 Jul 5;4:1181998. doi: 10.3389/fcdhc.2023.1181998 (PMC10354430; doi:10.3389/fcdhc.2023.1181998)
Supplement: Supplementary file 1 [file Table_1.docx]

**Supplementary Table 1** Characteristics of patients receiving or not receiving psychotropic drugs’ prescriptions.

| Characteristics | With psychotropic drugs | Without psychotropic drugs | p |
| --- | --- | --- | --- |
| N | 379 | 903 | - |
| Age (years) | 60.1 (14.4) | 65.4 (13.4) | <0.001 |
| Gender (male/female) | 179/200 | 530/373 | <0.001 |
| BMI (kg/m^2^) | 26.9 (6) | 24.9 (5.1) | <0.001 |
| Duration of diabetes (years) | 10.3 (10.3) | 12.3 (11.3) | 0.003 |
| Smoking habits (Brinkman index) | 298.9 (452.3) | 340.1 (584.7) | 0.17 |
| Drinking habits (g/day in ethanol consumption) | 14.5 (26.7) | 20.7 (34.6) | 0.001 |
| Exercise time (min/day) | 9.5 (33.1) | 18.8 (50.1) | <0.001 |
| Sleep duration (h) | 7.8 (2.1) | 7 (1.6) | <0.001 |
| Systolic blood pressure (mmHg) | 129.2 (18.5) | 131.9 (20.3) | 0.021 |
| Diastolic blood pressure (mmHg) | 73.2 (14.3) | 73.7 (14.1) | 0.53 |
| HbA1c (%) | 7.1 (1.4) | 7.7 (1.8) | <0.001 |
| eGFR (mL/min/1.73m^2^) | 75.2 (22.6) | 71.9 (23.9) | 0.023 |
| Handgrip strength (kg) | 22.3 (9.7) | 24.6 (9.6) | <0.001 |

Data are represented as the mean value (SD) except for the number of subjects and sex. BMI, body mass index; HbA1c, hemoglobin A1c; eGFR, estimated glomerular filtration rate.
