# Supplementary material for: Association of the use of psychotropic drugs with hospitalization, cardiovascular events, and mortality in patients with type 2 diabetes: a propensity score-matched cohort study
Source: Front Clin Diabetes Healthc. 2023 Jul 5;4:1181998. doi: 10.3389/fcdhc.2023.1181998 (PMC10354430; doi:10.3389/fcdhc.2023.1181998)
Supplement: Supplementary file 2 [file DataSheet_1.docx]

## Sample size calculation

Sample size calculation was performed using G*Power (https://www.psychologie.hhu.de/arbeitsgruppen/allgemeine-psychologie-und-arbeitspsychologie/gpower). To the best of our knowledge, there have been no systematic reviews and meta-analyses that examine the association between the use of psychotropic drugs and all-cause hospitalization; however, according to the study by Sun et al. (14) that examines the association between the use of benzodiazepines and benzodiazepine related drugs and the risk of pneumonia, effect size was set at 0.25, and one-tailed alpha level was 0.05. The sample size calculation indicates that 208 observations are required in each group for a power of 0.8. This suggests that our sample size had sufficient power to detect statistical significance.

**Reference**

14. Sun GQ, Zhang L, Zhang LN, Wu Z, Hu DF. Benzodiazepines or related drugs

and risk of pneumonia: a systematic review and meta-analysis. Int. J. Geriatr. Psychiatry (2019) 34:513–21. doi: 10.1002/gps.5048
